# A Role for High Mobility Group Box 1 (HMGB1) Release in the Pathogenesis of Gastroesophageal Reflux Disease

**DOI:** 10.1111/nmo.70083

**Published:** 2025-05-20

**Authors:** Tom Leech, David Kelsell, Diana Blaydon, Philip Woodland, Madusha Peiris

**Affiliations:** ^1^ Wingate Institute of Neurogastroenterology, Blizard Institute, the Faculty of Medicine and Dentistry Queen Mary University of London London UK; ^2^ Centre for Cell Biology and Cutaneous Research, Blizard Institute, the Faculty of Medicine and Dentistry Queen Mary University of London London UK

**Keywords:** gastroesophageal reflux disease, HMGB1, mucosa, oxidative stress

## Abstract

**Background:**

Many gastroesophageal reflux disease (GORD) patients have heartburn symptoms despite PPI treatment, potentially via esophageal mucosa exposure to weakly acidic bile salts, leading to oxidative stress. High mobility group box 1 (HMGB1) is a nuclear protein secreted during oxidative stress that binds to receptors including TLR2, TLR4, and RAGE, inducing inflammatory signaling. We describe the release and potential downstream effects of HMGB1 in GORD.

**Methods:**

Esophageal biopsies were obtained from healthy controls (HC), functional heartburn (FH), non‐erosive reflux disease (NERD), and erosive reflux disease (ERD) patients. Biopsies were analyzed by RNA‐sequencing, and the expression of TLR2, TLR4, and RAGE was assessed with immunohistochemistry. NE‐1 cells were challenged with pH 5 media and/or 500 μM deoxycholate (DCA), with/without 2‐h 5 or 50 μM curcumin pre‐treatment. HMGB1 translocation was measured with immunofluorescence and release with ELISA.

**Results:**

HMGB1, alongside 12 additional oxidative stress‐associated genes, is over‐expressed in NERD and ERD patients compared to HC (adjusted *p* < 0.05). Weakly acidic bile salt (pH 5 + DCA) stimulated HMGB1 translocation (*p* < 0.0001) and release from NE‐1 cells (*p* < 0.001), but ameliorated with curcumin pre‐treatment. ERD biopsies had increased DAPI+TLR4+ cells (*p* < 0.05) and TLR4 fluorescence intensity (*p* < 0.05), compared to HC. RAGE was expressed on CD45+ cells, and the number of RAGE+CD45+ cells was higher in ERD compared to HC (*p* < 0.05).

**Conclusions:**

Release of HMGB1 from esophageal epithelial cells by weakly acidic bile salts, inhibited by curcumin, and increased expression of TLR4 and RAGE in ERD mucosa suggest this pathway has a role in GORD. Modulating HMGB1 activity may be a strategy for treating recurrent symptoms.

## Graphical abstract

Incubation of esophageal epithelial cells with a weakly acidic (pH 5) bile salt (deoxycholic acid) induces translocation of HMGB1 from the nucleus to the cytosol, and its subsequent release from the cell. This process can be ameliorated by pre‐treatment with the antioxidant, curcumin. The HMGB1 receptors, TLR2, TLR4, and RAGE, are present in the esophageal mucosa of healthy subjects and gastro‐esophageal reflux disease (GORD) patients, on epithelial cells (TLR2 & TLR4) and CD45+ immune cells (RAGE). Created with BioRender.com

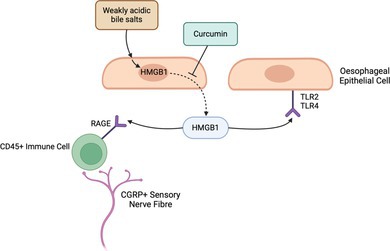

**Summary**

- Oxidative stress‐associated genes are overexpressed in the esophageal mucosa of both non‐erosive and erosive reflux disease patients, including HMGB1.
- HMGB1 is released from NE‐1 esophageal epithelial cells in vitro in response to weakly acidic bile salts; however, pre‐treatment with curcumin, an antioxidant, ameliorates HMGB1 release.
- Receptors that are activated by HMGB1 are expressed on epithelial cells and immune cells in human esophageal mucosa.

**Abbreviations**
AOI: area of interest
BO: Barrett's esophagus
CGRP: calcitonin gene‐related peptide
DCA: deoxycholic acid
ERD: erosive reflux disease
FH: functional heartburn
GORD: gastroesophageal reflux disease
HMGB1: high mobility group box 1
IHC: immunohistochemistry
mRNA: messenger ribonucleic acid
NADPH: nicotinamide adenine dinucleotide phosphate
NERD: non‐erosive reflux disease
PPI: proton pump inhibitor
RAGE: receptor for advanced glycation endproducts
ROS: reactive oxygen species
TLR: toll‐like receptor

## 1 Introduction

Gastroesophageal reflux disease (GORD) is a chronic condition resulting from the retrograde movement of refluxate from the stomach into the esophagus. GORD affects approximately 20%–25% of UK adults, and global prevalence has increased steadily over the last 20 years [1]. Heartburn is a form of visceral pain and results from the activation of esophageal sensory afferent nerves by luminal refluxate exposure or inflammatory components produced within the mucosa [2, 3]. Patients with GORD have impaired esophageal barrier function, as well as inflammation or micro‐inflammation of the mucosa, resulting in the activation of sensory nerve fibers that lead to visceral pain and neuronal sensitisation [3, 4, 5, 6, 7]. Although endoscopically visible esophageal inflammation (oesophagitis) is a common finding in GORD (erosive reflux disease (ERD)), the majority of patients—estimates range from 50% to 80%—lack endoscopically visible esophageal inflammation (non‐erosive reflux disease (NERD)), but have markers of mucosal micro‐inflammation such as leukocyte infiltration, epithelial production of cytokines, and loss of barrier function [8, 9]. Increasing evidence suggests that neuro‐immune crosstalk in both micro‐ and macro‐inflammation in GORD may contribute to heartburn symptoms in patients, making the investigation of these mechanisms a promising therapeutic target [10].

Proton pump inhibitors (PPIs) are the first‐line treatment for GORD, and a recent systematic review found that 23% of the studied patient population were PPI users [11]. However, PPIs are insufficiently effective in reducing heartburn symptoms in up to 40% of GORD patients [12]. PPI‐refractory GORD is more common in NERD than ERD, suggesting that heartburn in this cohort is the result of factors other than luminal acid exposure [13]. There are multiple potential causes underlying PPI‐refractory GORD, including poor compliance with medication and hypervigilance to esophageal discomfort [12]. However, non‐acid components of refluxate, such as bile salts, may also play a role in PPI‐refractory heartburn symptoms. Indeed, PPI‐treated NERD patients with abnormally high bile reflux report significantly more heartburn symptoms than those with physiological levels of bile reflux [14]. Further, esophageal perfusion of bile salts, even at neutral pH, induces pain symptoms [15]. A systematic review of the activity of bile salts on the esophageal mucosa in vitro and ex vivo concluded that bile salts cause esophageal epithelial cells to produce inflammatory mediators and induce an oxidative stress response [16]. In epithelial cells of the GORD esophagus, this oxidative stress may be the result of a build‐up of reactive oxygen species (ROS) due to NADPH oxidase activity [17]. Therefore, oxidative stress and inflammation due to esophageal reflux of bile salts in a weakly acidic environment may represent a target for the treatment of GORD, including PPI‐refractory GORD.

HMGB1 is a highly abundant non‐histone nuclear protein with physiological functions including chromosomal regulation [18]. However, during conditions of cellular stress, particularly a build‐up of ROS leading to oxidative stress, HMGB1 is acetylated at its nuclear localisation sequence, preventing sequestering to the nucleus [19]. Cytosolic HMGB1 is subsequently actively released via secretory lysosomes into the extracellular space via intracellular conditions such as ROS‐induced cellular stress or NF‐κB signaling [19]. Once in the extracellular space, HMGB1 acts as a cytokine and has activity at various receptors, including TLR2, TLR4, and RAGE [18, 20, 21]. TLR2 and TLR4 are located on epithelial cells in the esophageal mucosa, and their expression is increased in esophageal squamous cell carcinoma (ESCC) as well as in oesophagitis [22, 23]. Expression of RAGE is low in the healthy esophageal mucosa, but is reported to be increased in ESCC [24]. Activation of these receptors by HMGB1 results in downstream activation of NF‐κB signaling pathways, leading to the production of inflammatory cytokines such as IL‐6, TNF, and IFN‐ [21]. In murine ileum, as well as Caco‐2, lung, and bronchial cells, HMGB1 signaling also increases epithelial permeability, allowing transmission of large molecules across the epithelium [25, 26]. Therefore, activation of these receptors on cells of the human esophagus by HMGB1 could drive inflammatory cytokine production, leading to a loss of barrier integrity and thus increasing epithelial permeability.

Therefore, we hypothesized that HMGB1 secretion as a result of oxidative stress in the esophageal mucosa in response to weakly acidic bile salts may be a possible mechanism contributing to inflammation in the GORD. We demonstrated the mechanism of HMGB1 release from the human esophagus in response to refluxate exposure. Secondly, we characterized the expression of HMGB1 receptors TLR2, TLR4, and RAGE in the esophageal mucosa of healthy volunteers and GORD patients to determine potential downstream impacts of HMGB1 release.

## 2 Methods

### 2.1 Study Subjects

All patients were prospectively recruited following informed consent and were required to have a clinical history of problematic heartburn requiring investigation. Criteria for inclusion: (1) aged between 18 and 70 years, (2) had symptoms of at least moderate heartburn more than three times per week, and (3) had a clinical referral for endoscopic examination for investigation of symptoms. Patients were excluded if they had: (1) previous upper gastrointestinal surgery, (2) severe upper gastrointestinal motility disorders, (3) coagulopathy or concurrent anticoagulant medication, (4) were pregnant, (5) were allergic or hypersensitive to local anesthetic, and (6) had any other medical condition that would make it unsafe for the subject to participate, as determined by the treating physician.

Patients underwent endoscopy ± wireless ambulatory reflux monitoring. All patients had stopped PPI treatment for at least 7 days before endoscopy and reflux testing. Post‐procedure, patients were divided according to phenotypes as follows: (1) erosive reflux disease (ERD), (2) nonerosive reflux disease (NERD), and (3) functional heartburn (FH) according to the definitions below. Patients with pathological acid exposure (> 6% over the study period) on analysis of their reflux studies qualified for a diagnosis of NERD. Patients whose reflux testing studies did not meet pathological acid exposure criteria and had negative reflux/symptom association were classified as FH. Symptomatic patients with at least LA grade B esophagitis at endoscopy were classified as ERD. These phenotypic classifications are supported by the Lyon Consensus 2.0 [27].

A total of 32 GORD patients included for immunohistochemical analysis in this study were recruited from the Royal London Hospital (Barts and the London School of Medicine and Dentistry, Queen Mary University of London, UK). This study was granted ethical approval by the NRES Committee London—Queen Square (Study reference: 19/LO/1506).

This study included ten asymptomatic volunteers. These volunteers had no history of gastrointestinal symptoms or anti‐reflux medication use and had a Reflux Disease Questionnaire score of 0. Healthy controls were excluded if they: (1) had previous upper GI surgery, (2) had severe upper GI motility disorders, (3) were pregnant, (4) were taking coagulopathy or concurrent anticoagulant medication, or (5) had any severe midface trauma or recent nasal surgery. All subjects had a normal esophageal appearance on endoscopy. Distal esophageal biopsies of these HCs were prepared and analyzed in an identical fashion to the patient biopsies used in this study.

### 2.2 Bulk RNA Sequencing Analysis

To identify oxidative stress–related genes which are upregulated in NERD and ERD esophagus compared to healthy controls, re‐analysis was performed on previous bulk RNA sequencing data collected by our group and published by Ustaoglu et al. [28]. Therefore, all samples from this dataset were phenotyped according to the criteria outlined above and included 8 healthy controls, 8 FH, 9 NERD, and 10 ERD patients [28]. Following previously reported methods [29], 1399 oxidative stress protein domains were extracted from GeneCards (https://www.genecards.org) with a relevance score ≥ 7 [30]. Differential expression analysis (DEseq2) was performed between healthy controls and FH patients, healthy controls and NERD patients, as well as healthy controls and ERD patients, with an FDR‐adjusted *p*‐value filter of < 0.05. Genes with a log_2_ fold change (FC) < 1 or an average transcripts per million (TPM) count of < 10 were removed to identify highly‐expressed oxidative stress–related genes which are overexpressed in both NERD and ERD. This sequencing data is deposited in the NCBI's Gene Expression Omnibus and is accessible through the GEO Series accession number GSE226303.

### 2.3 Cell Culture and Treatment

NE‐1 cells (AddexBio, Cat. Number T0013001) were cultured in Keratinocyte SFM (KSFM; Gibco, Cat. Number 17005042) supplemented with 0.4% Penicillin/Streptomycin (50 U/mL; Gibco, Cat. Number 15140122) in 5% CO_2_ at 37°C. Cells between passages 16–20 were seeded at a concentration of 2 × 10^5^ cells/well or 2 × 10^4^ cells/well into 12‐well plates and 8‐well Lab‐Tek chamber slides (Thermo Scientific, Cat. Number 177429), respectively. Cells were incubated for 2 days, and media were changed each day prior to the assay.

Cells were pre‐treated with warm KSFM, 5 μM, or 50 μM curcumin in KSFM for 2 h. Wells were rinsed twice with warm KSFM to remove excess curcumin. Wells were treated for 10 min with either: (1) pH 7 KSFM, (2) pH 5 KSFM, (3) pH 7 KSFM + 500 μM deoxycholate (DCA), (4) pH 5 KSFM + 500 μM DCA. Incubation of esophageal epithelial cells with DCA in an acidic medium induces ROS production on this timescale and was therefore chosen [17, 31].

### 2.4 HMGB1 Immunocytochemistry

Following treatment, cells were immediately rinsed with ice‐cold PBS and permeabilized with 0.1% Triton X‐100 in PBS for 20 min. Cells were rinsed three times in PBS and covered with 3% BSA in PBS for 30 min before being incubated overnight at 4°C with primary antibody solution containing HMGB1 (1:400 dilution; monoclonal rabbit anti‐human; Abcam, Cat. Number Ab79823) and E‐Cadherin (1:300 dilution; monoclonal mouse anti‐human; Invitrogen, Cat. Number 13‐1700). Cells were washed three times in PBS and incubated for 1 h with the secondary antibody (donkey anti‐mouse 568 nm; donkey anti‐rabbit 568 nm; Invitrogen, Thermo Fisher Scientific; 1:400 dilution). Cells were washed three times in PBS and mounted with a coverslip with antifade mounting medium containing a DAPI fluorescent stain (Vector Laboratories, Cat. Number H‐1500). Slides were examined for fluorescence using a Leica DM5000B Epi‐Fluorescence Microscope, and images were captured using MetaMorph software.

Images were captured with a 40× lens and processed using FIJI software. A minimum of 3 images were captured per well using DAPI as a positional marker. Areas of interest (AOIs) were drawn around the nuclei and cell membranes, using DAPI and E‐Cadherin staining, of each cell in the field of view (FOV). HMGB1 fluorescence was measured in the nuclei and cytosol of each cell and used to produce a ratio of cytosol: nucleus fluorescence intensity. Each data point represents the mean cytosolic: nuclear ratio of each cell analyzed per well.

### 2.5 HMGB1 Release Assay

Cells were pre‐treated and challenged as described previously; however, the challenge period was 20 min to allow measurable release of HMGB1. After 20 min, the media were removed and stored at −80°C prior to analysis. The concentration of HMGB1 in the supernatant was analyzed by ELISA (Novus Biologicals, Cat. Number NBP2‐62781).

### 2.6 CellROX Assay

Cells were seeded on a 24‐well plate at a concentration of 1 × 10^5^ cells/well. Cells were incubated for 2 days, and media were changed each day prior to the assay. Cells were pre‐treated with pre‐warmed KSFM or 50 μM curcumin in KSFM for 2 h. Wells were rinsed twice with warm KSFM to remove excess curcumin. Cells were treated for 10 min with: (1) pH 7 KSFM, (2) pH 5 KSFM, (3) pH 7 KSFM + 500 μM deoxycholate (DCA), or (4) pH 5 KSFM + 500 μM DCA. Wells were rinsed twice with KSFM and incubated for 30 min with 5 μM CellROX in KSFM (Invitrogen, Cat. Number C10422). Wells were rinsed three times with PBS and fixed with 3.7% PFA for 15 min at room temperature, in the dark. Wells were rinsed twice with PBS, and images were captured within 1 h. Images were taken at 20× using an Olympus IX83 Microscope and were captured using Olympus cellSens software. CellROX fluorescence was captured at a wavelength of 647 nm, and the cell outline was determined using phase contrast. The experiment was performed 3 times, and at least 2 wells were used each time as technical replicates. For each well, a minimum of 3 images were captured, and 20–30 cells per image were selected at random; a region of interest (ROI) was drawn around the cell using FIJI to calculate mean fluorescence intensity within the ROI.

### 2.7 Total Antioxidant Capacity

Total antioxidant capacity was measured in NE‐1 cells treated with curcumin. 6 × 10^5^ NE‐1 cells were seeded onto wells (6‐well plate), and the media was changed after 24 h. After 48 h, the media were removed and cells were incubated with KSFM or 50 μM curcumin for 2 h. After 2 h, cells were mechanically lysed in 150 μL H_2_O, and total antioxidant capacity was determined using a commercially available kit according to the manufacturer's instructions (Abbexa, Cat. Number abx295022). This assay was performed 3 times, and data are presented as mean ± standard deviation (SD) fold‐change compared to control.

### 2.8 Immunohistochemistry of HMGB1 Receptors in Esophageal Mucosa

Biopsies were immediately fixed in 4% PFA (Sigma‐Aldrich, Cat. Number 158127) in phosphate‐buffered saline for 2 h. Biopsies were washed three times in PBS and placed in 30% sucrose in PBS for 24 h at 4°C. Fixed tissue was embedded in optimum cutting temperature compound (OCT compound; Sakura Tissue‐Tek, Cat. Number 4583) and frozen at −20°C. Serial 10 μm sections were cut and mounted on positively charged glass slides (Thermo Scientific, Cat. Number J1800AMNZ).

For immunohistochemistry (IHC), slides were washed with PBS for 5 min to rehydrate the sections, before being blocked with protein block (Protein Block Serum‐Free Ready‐to‐use, Dako, Cat. Number X0909) for 1 h. Sections were incubated with primary antibody in PBS with 0.2% Triton X‐100 for 16–18 h at 4°C. Antibodies used were: E‐Cadherin (1:300 dilution; monoclonal mouse anti‐human; Invitrogen, Cat. Number 13‐1700), TLR2 (1:200 dilution; polyclonal rabbit anti‐human; Abcam, Cat. Number Ab191458), TLR4 (1:150 dilution; polyclonal rabbit anti‐human; Proteintech, Cat. Number 19811‐1‐AP), RAGE (1:200 dilution; polyclonal rabbit anti‐human; Abcam, Cat. Number Ab37647), CD45 (1:100 dilution; monoclonal mouse anti‐human; Dako, Cat. Number M0701), CGRP (1:150 dilution; monoclonal mouse anti‐human; Thermo Fisher, Cat. Number 025‐05‐02). Slides were washed in PBS three times and incubated with 1:400‐diluted secondary antibody (donkey anti‐mouse 568 nm; donkey anti‐rabbit 568 nm; Invitrogen, Thermo Fisher Scientific; 1:400 dilution) at room temperature for 1 h. Slides were then washed in PBS three times and mounted with a coverslip with antifade mounting medium containing a DAPI fluorescent stain (Vector Laboratories, Cat. Number H‐1500).

A minimum of 3 fields of view (FOVs) per sample were captured, and staining of TLR2 and TLR4 was quantified in a semi‐automated manner. An AOI was drawn manually around the esophageal epithelium, and the mean staining intensity of TLR2 or TLR4 in the AOI was measured using ImageJ (U.S. National Institutes of Health, USA). For cell counts of TLR2+ and TLR4+ cells, image files were named to minimize bias, and cells with positive staining were counted. The channel containing DAPI staining was converted to binary, and the number of nuclei was quantified. To count RAGE+ cells, at least 3 FOVs were captured per patient, at esophageal papillae, where the numbers of CD45+ cells are highest. The mean number of CD45+RAGE+ cells per FOV was calculated for each patient.

### 2.9 Statistical Analysis

One‐way ANOVA tests were used to analyze differences between groups. When ANOVA was positive, Tukey's multiple comparisons test was used to identify which of the pairs was significantly different. To analyze differences between RAGE+ cell counts of groups, the non‐parametric Kruskal–Wallis test with Dunn's post hoc test was used. An unpaired *t*‐test was used to analyze differences in total antioxidant capacity between control and 50 μM curcumin‐treated cells. Values are expressed as mean ± standard deviation (SD). GraphPad Prism 10 was used for statistical analysis. Patient demographics are shown in Table 1.

**TABLE 1:** Patient demographics and sample numbers per analysis.

Phenotype	Number of patients	Mean age (years)	Age range (years)	Female:male
Bulk RNA‐sequencing samples				
HC	8	27	25–55	6:2
FH	8	41	20–72	3:5
NERD	9	44	29–55	3:6
ERD	10	46	22–73	2:8
TLR4 immunofluorescence samples				
HC	10	30	20–70	8:2
FH	7	38	20–72	2:5
NERD	13	47	27–69	7:6
ERD	11	44	22–55	2:9
TLR2 and RAGE immunofluorescence samples				
HC	9	31	25–70	7:2
FH	5	39	20–72	1:4
NERD	12	47	27–69	6:6
ERD	13	47	22–74	8:5

## 3 Results

### 3.1 HMGB1 Is Overexpressed in the Esophageal Mucosa of NERD and ERD Patients

To determine whether oxidative stress pathways are significantly activated in GORD, and the downstream intracellular impacts of oxidative stress in the esophagus in GORD phenotypes, bulk RNA sequencing data were analyzed from esophageal mucosal biopsies of healthy controls, functional heartburn, non‐erosive reflux disease, and erosive reflux disease patients [28].

**FIGURE 1:**
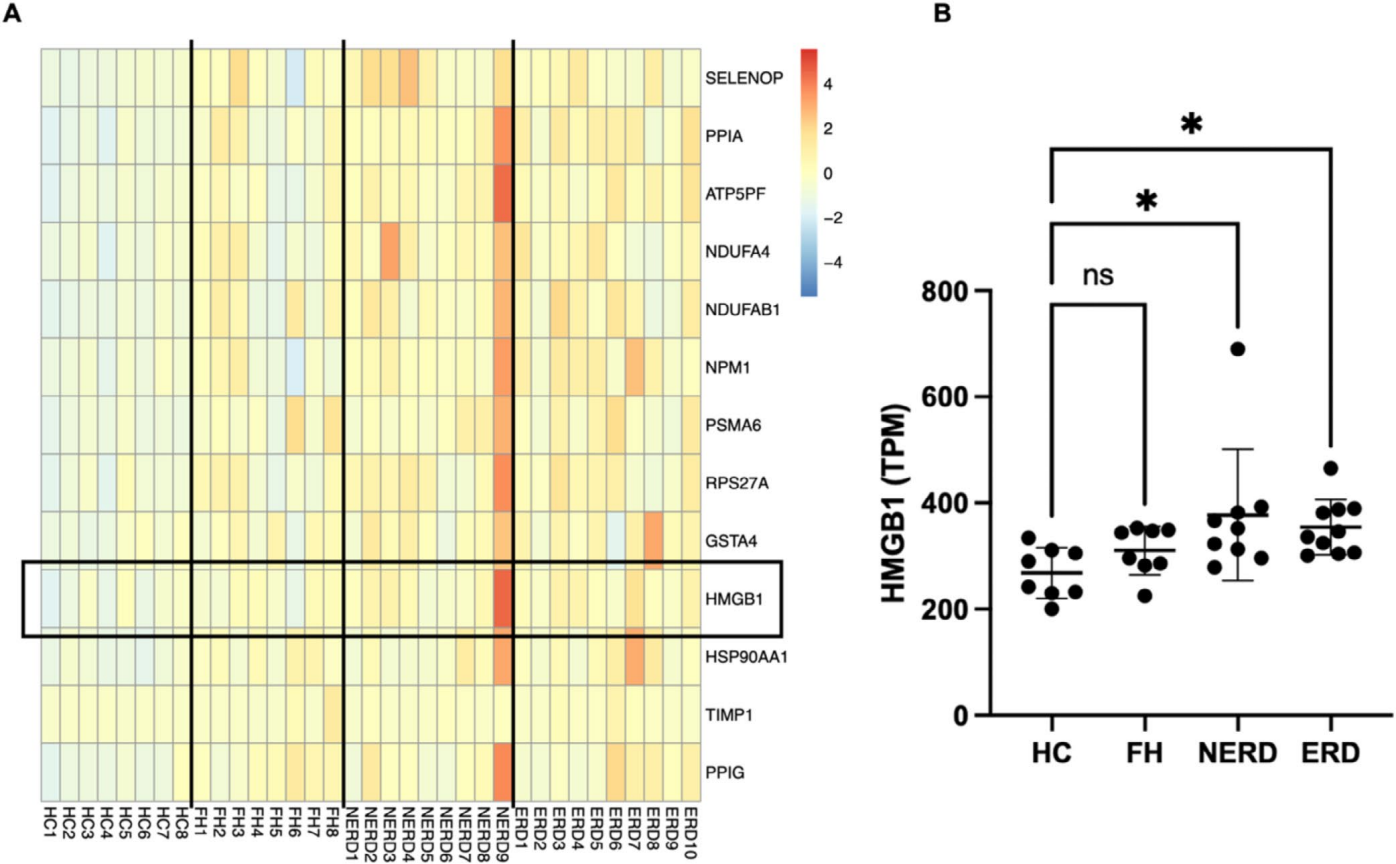
HMGB1 is one of the 13 highly expressed oxidative stress‐associated genes which was upregulated in both NERD and ERD. 1399 oxidative stress protein domains were extracted from GeneCards (https://www.genecards.org) with a relevance score ≥ 7. Differential expression analysis (DEseq2) was performed between healthy controls and FH patients, healthy controls and NERD patients, as well as healthy controls and ERD patients, with an FDR‐adjusted *p* value filter of < 0.05. Genes with a log_2_ fold change (FC) < 1 or an average transcripts per million (TPM) count of < 10 were removed (A) A heatmap of the 13 oxidative stress ‐associated genes with average TPM > 10 which are upregulated in both NERD and ERD following bulk RNA sequencing of healthy controls (HC), functional heartburn (FH), non‐erosive reflux disease (NERD), and erosive reflux disease (ERD) patients (*n* = 8–10). (B) A graph showing HMGB1 mRNA expression in bulk RNA sequencing is significantly increased in NERD (FDR adj. *p* = 0.026) and ERD (FDR adj. *p* = 0.00098) esophageal mucosa.

A total of 1399 oxidative stress–associated genes were identified and analyzed [30]. After filtering out lowly expressed genes (mean TPM < 10), 775 genes remained for FH, 776 for NERD, and 769 for ERD. Compared to healthy controls, 1 of these genes was found to be highly expressed (mean TPM > 10) and upregulated in FH (RNASET2), 26 genes in NERD, and 43 genes in ERD (FDR adj. *p* < 0.05; Table S1). Of these genes, 13 were overexpressed in both NERD and ERD esophageal mucosa compared to healthy controls (Figure 1A). None of these genes were upregulated in FH. Several of these genes encode proteins involved in cellular processes, such as ribosome biogenesis (RPS27LA, NPM1), proteasome formation (PSMA6), and protein folding (PPIG). Three of these genes (*ATP5PF, NDUFA4, NDUFAB1*) encode proteins involved in oxidative phosphorylation. Several other upregulated genes may be relevant to GORD pathogenesis, including the metalloproteinase inhibitor, *TIMP1*, the Glutathione S‐transferase enzyme, *GSTA4*, the extracellular antioxidant, *SELENOP*, the heat shock protein, *HSP90AA1*, and the pro‐apoptotic protein, *PPIA*. However, these genes do not have well‐defined links to oxidative stress‐induced mucosal inflammation.

*HMGB1* was found to be expressed more highly in both NERD (FDR adj. *p* = 0.026) and ERD (FDR adj. *p* = 0.00098), but not FH (FDR adj. *p* = 0.18), compared to healthy controls (Figure 1B). HMGB1 is a nuclear protein that is released from cells in conditions of oxidative stress; its release and subsequent paracrine induction of inflammatory pathways via receptor activation have been implicated in several inflammatory conditions in the GI tract, skin, and other tissues [32].

### 3.2 HMGB1 Is Released From Esophageal Epithelial NE‐1 Cells in Response to Weakly Acidic Bile Salts

To predict its role in GORD, we determined HMGB1 release from esophageal epithelial cells in response to mixtures of weak acid and bile salt to simulate refluxate. To assess whether HMGB1 is translocated from the nucleus to the cytosol in response to weakly acidic bile salts, we treated NE‐1 cells for 10 min with combinations of the bile salt deoxycholic acid (DCA) and weakly acidic (pH 5) media.

DCA has been detected in the gastric juice of healthy individuals [33], and the relative concentration of unconjugated bile salts, such as DCA, may be higher in the gastric juice of patients receiving PPIs [34] and individuals with high levels of bile reflux [35]. The ability of DCA at a weakly acidic pH (pH 4–6) to induce an oxidative stress response in esophageal epithelial cells has been extensively corroborated [16, 33]. E‐Cadherin was used as a marker to stain the outer cell membrane [36].

**FIGURE 2:**
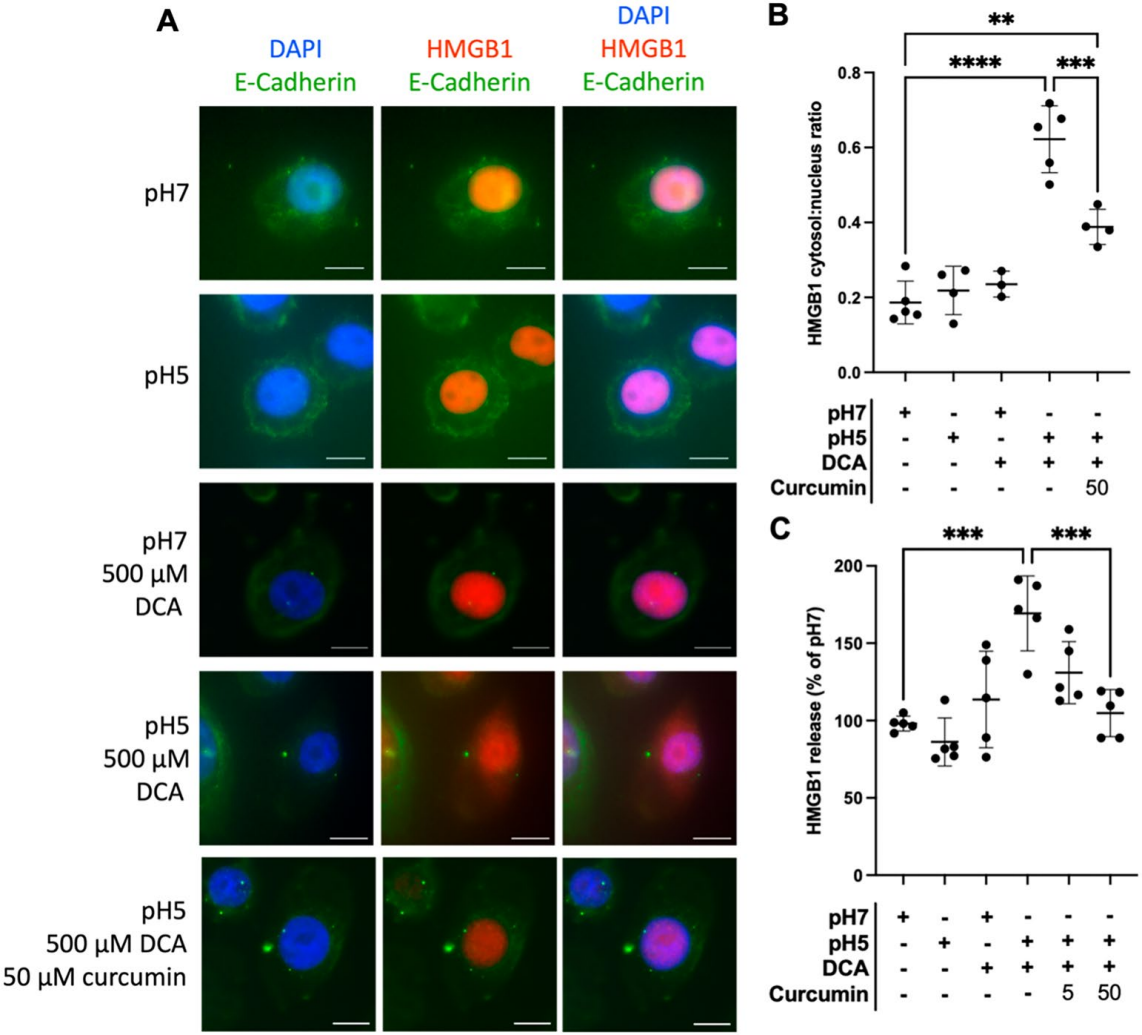
NE‐1 esophageal epithelial cells were pre‐treated with KSFM or 50 μM curcumin for 2 h. Wells were rinsed twice with KSFM to remove excess curcumin. Wells were treated for 10 min (HMGB1 translocation) or 20 min (HMGB1 release) with either (1) pH 7 KSFM, (2) pH 5 KSFM, (3) pH 7 KSFM +500 μM deoxycholate (DCA), (4) pH 5 KSFM +500 μM DCA. (A) Representative images showing translocation of HMGB1 from the nucleus in cells treated with acidic bile salts. (B) The ratio of cytosolic: Nuclear HMGB1 as measured by fluorescence intensity (*n* = 3–5). (C) Release of HMGB1 from NE‐1 esophageal epithelial cells following 20 min of treatment with combinations of acid and bile salts (*n* = 5). Scale bar = 20 μm. Mean ± SD. Images were chosen representatively from 5 independent experiments. Images were captured with a Leica DM5000B Epi‐Fluorescence Microscope and a DFC350 FX camera using MetaMorph Software. Quantification of protein expression or staining was performed using ImageJ (National Institutes of Health). **p < 0.01, ***p < 0.001, ****p < 0.0001.

HMGB1 expression was restricted to the nucleus in control cells, as well as cells treated with either acid or DCA alone. However, a combination of weak acid and bile salt (pH 5 + 500 μM DCA) resulted in cytosolic expression of HMGB1 with a diffuse staining pattern observed across the cellular environment (Figure 2A). Indeed, only the combination of weak acid and DCA caused an increase in the cytosolic: nuclear ratio of HMGB1, representing loss of HMGB1 from the nucleus of the cells (0.187 ± 0.057 vs. 0.622 ± 0.089; *p* < 0.0001; Figure 2A,B).

To quantify HMGB1 release from NE‐1 cells in response to challenge with acid and DCA, we measured HMGB1 in the supernatant following a 20‐min challenge. This revealed a similar pattern, with acid or DCA alone not significantly altering HMGB1 release compared to control media. HMGB1 release from NE‐1 cells challenged with pH 5 media containing 500 μM DCA for 20 min was, however, higher than that from cells exposed to pH 7 media (24.2 pg/mL ± 6.93 vs. 42.6 pg/mL ± 18.6; *p* < 0.001; Figure 2C).

HMGB1 translocation from the nucleus and subsequent release can be due to intracellular oxidative stress [18]. To prevent an oxidative stress response in NE‐1 cells following challenge with weakly acidic bile salts, cells were pre‐treated with 50 μM curcumin, a turmeric extract with antioxidant properties [37], for 2 h prior to the challenge. Curcumin pre‐treatment significantly decreased the cytosolic:nuclear HMGB1 ratio, observed as greater nuclear expression of HMBG1 with reduced diffuse translocation into the cytosol (0.622 ± 0.089 vs. 0.3881 ± 0.047 *p* < 0.001; Figure 2A,B). Similarly, pre‐treatment with curcumin ameliorated HMGB1 release from NE‐1 cells following 20 min of incubation with pH 5 DCA (42.61 ± 18.6 vs. 30.93 ± 5.36; *p* < 0.05; Figure 2C).

To directly demonstrate the effect of curcumin treatment in reducing oxidative stress in NE‐1 cells following weakly acidic bile salt treatment, oxidative stress was detected further via CellROX staining. Treatment with pH 5 + DCA, but not pH 5 or DCA alone, significantly increased the mean CellROX fluorescent intensity in NE‐1 cells compared to control (*p* < 0.0001; Figure S1B). Curcumin pre‐treatment reversed the increase in oxidative stress (*p* = 0.0005; Figure S1B). In addition, the total antioxidant capacity of curcumin pre‐treated cells increased 4.5‐fold compared to control cells (*p* = 0.0103; Figure S1C).

### 3.3 TLR2 and TLR4 Are Present on Esophageal Epithelial Cells of Healthy Controls and GORD Patients

**FIGURE 3:**
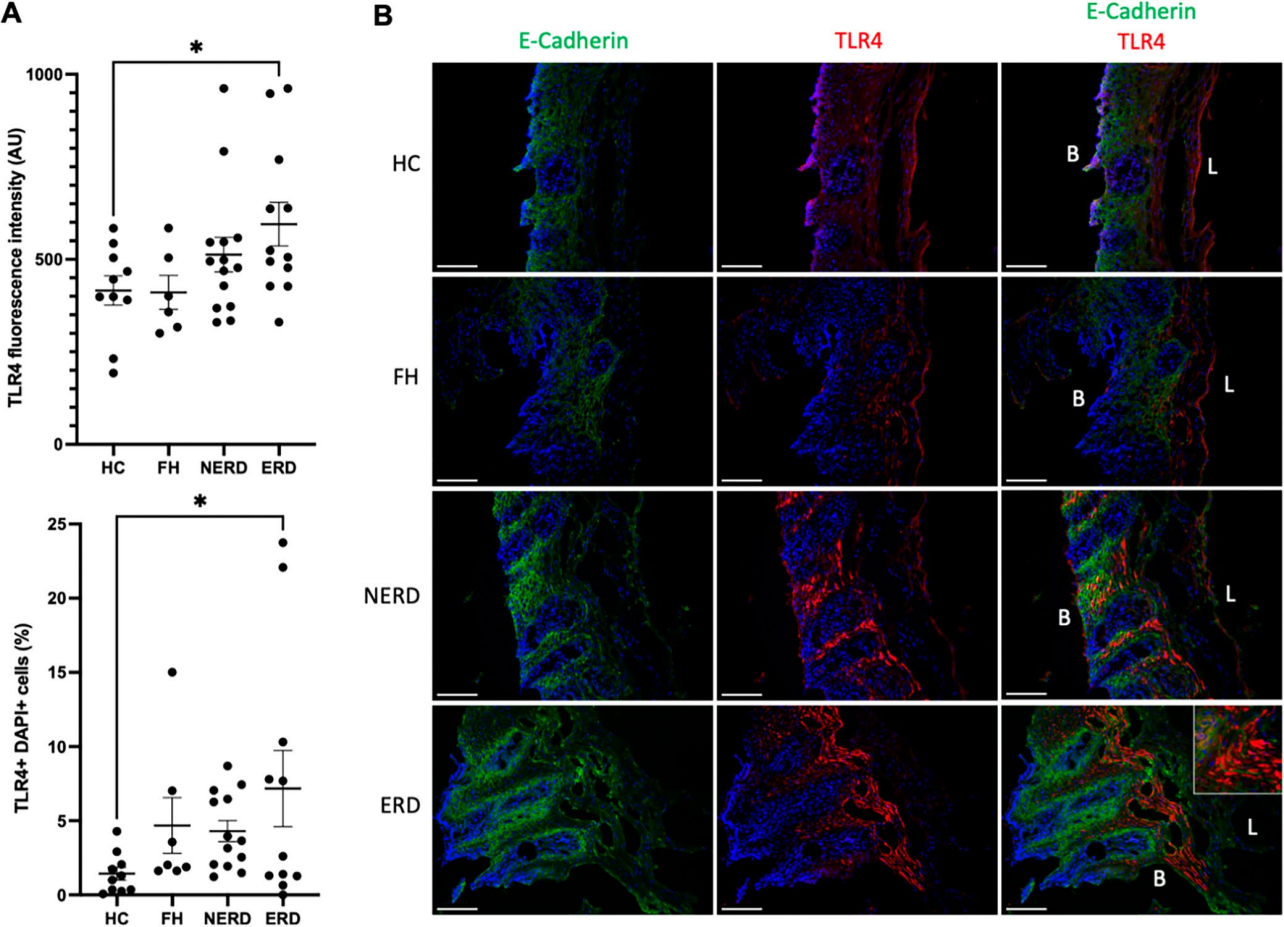
Quantification of TLR4 in the esophagus of healthy controls and GORD patients. (A) Mean fluorescence intensity of TLR4 signal in the esophageal epithelium and % of DAPI+ cells that were TLR4+. (B) Representative images of TLR4 in the esophageal mucosa. Mean ± SD. L = luminal side of epithelium. B = basal side of the epithelium. Images were captured with a Leica DM5000B Epi‐Fluorescence Microscope and a DFC350 FX camera using MetaMorph Software. Quantification of protein expression or staining was performed using ImageJ (National Institutes of Health). Scale bar = 200 μm. *p < 0.05.

To investigate the downstream impact of HMGB1 release from esophageal epithelial cells in response to weakly acidic reflux, we investigated the expression of its receptors, TLR2 and TLR4, in the esophageal mucosa of healthy controls and GORD patients. TLR4 was expressed on epithelial cells of the esophagus, and the fluorescence intensity as well as the % of TLR4+ DAPI+ cells were higher in the esophageal mucosa of ERD patients compared to healthy controls (*p* < 0.05; Figure 3A,B). TLR4 was generally expressed in the most luminal third of the esophageal epithelium; however, in GORD patients, protein expression extended towards the basal side (Figure 3A). TLR2 was also expressed on epithelial cells of the esophagus in both healthy controls and GORD patients, and TLR2 fluorescence intensity was unchanged between patient phenotypes (Figure S2).

### 3.4 RAGE Is Expressed on Immune Cells in the Esophageal Mucosa

**FIGURE 4:**
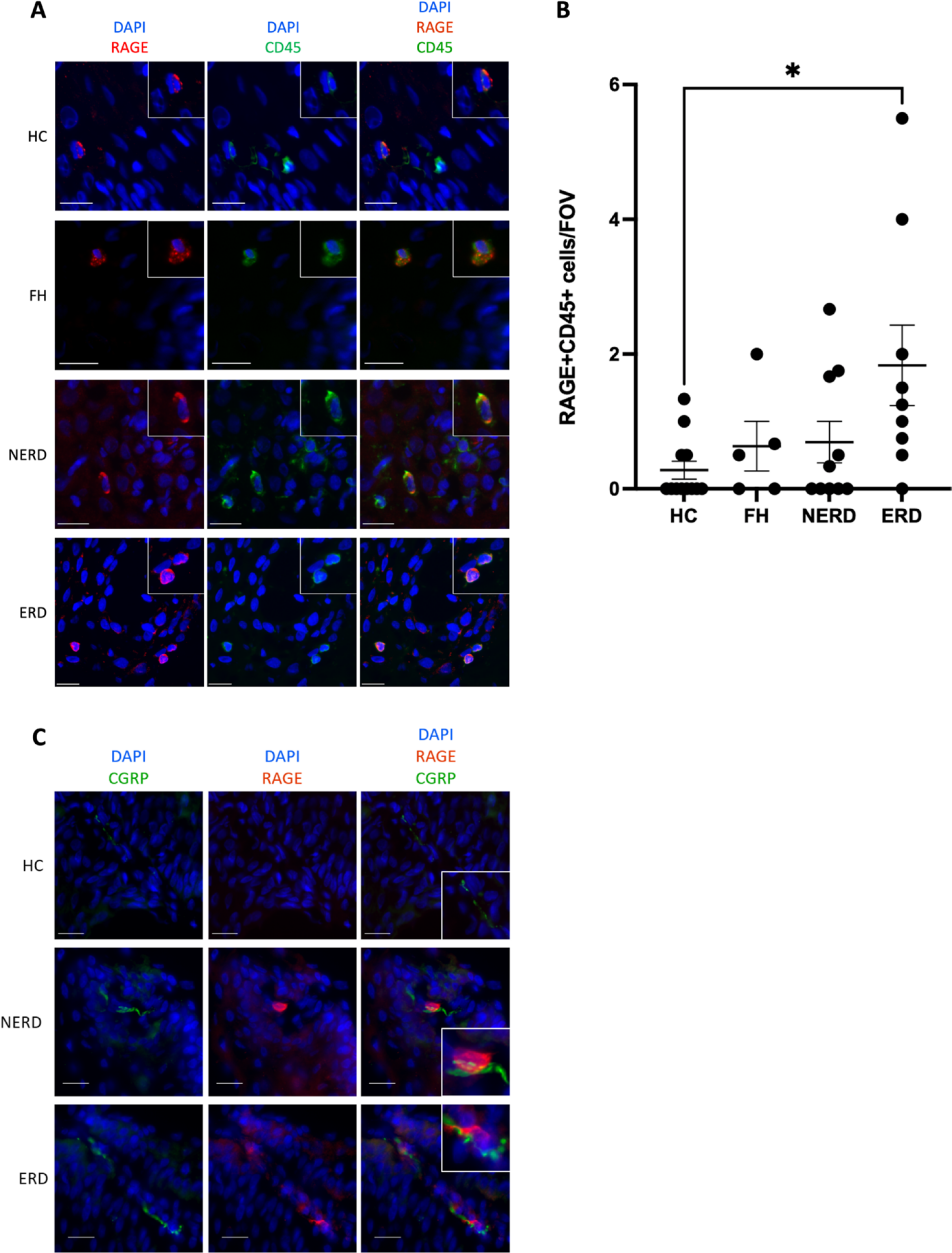
(A) CD45+ RAGE+ leukocytes were observed in the esophageal mucosa, particularly in NERD and ERD patients. (B) Quantification of RAGE+ CD45+ immune cells in the esophageal mucosa of GORD patients. Mean ± SD (C) Several RAGE+ cells were closely apposed to CGRP+ nerve fibers in papillae of the esophageal mucosa of NERD and ERD patients. Mean ± SD. Images were captured with a Leica DM5000B Epi‐Fluorescence Microscope and a DFC350 FX camera using MetaMorph Software. Quantification of protein expression or staining was performed using ImageJ (National Institutes of Health). Scale bar = 20 μm. *p < 0.05.

Activation of RAGE, a receptor for HMGB1, results in intracellular inflammatory signaling [38]. To determine if RAGE was associated with HMGB1 signaling in esophageal mucosa, we assessed its localisation in esophageal biopsy tissue. In our study, RAGE was expressed exclusively on CD45+ immune cells in the mucosa of healthy controls and GORD patients (Figure 4A). RAGE+CD45+ cells were present primarily within the mucosal papillae, although many intra‐epithelial RAGE+CD45+ cells were observed. Although RAGE+CD45+ cells were rare within the esophageal mucosa of all subjects, there were significantly more in ERD mucosa compared to healthy controls (Figure 4B; *p* < 0.05).

To determine the proximity of these RAGE+ immune cells to sensory nerve fibers, RAGE was co‐stained with the sensory neuronal marker, CGRP [39]. CGRP+ nerve fibers were observed as pearl‐like staining in papillae of the esophageal mucosa, which is typical of nerve fibers in the esophageal mucosa [5]. In both NERD and ERD patients, a proportion of RAGE+ cells were in close apposition to CGRP+ nerve fibers in papillae of the esophageal mucosa (Figure 4C).

## 4 Discussion

We have demonstrated that exposure of esophageal epithelial cells to DCA in a weakly acidic medium promotes the release of the inflammatory protein, HMGB1, and that this can be ablated by pre‐treatment with curcumin. This pathway is relevant to both NERD and ERD pathogenesis as HGMB1 is overexpressed in both disease phenotypes. The presence of HMGB1 receptors, TLR2, TLR4, and RAGE in the esophageal mucosa represents paracrine targets of epithelial‐released HMGB1 in driving mucosal inflammation and potentially neuronal sensitization through close interactions with sensory afferent nerve fibers.

Oxidative stress is a key pathway leading to translocation of HMGB1 from the nucleus and its subsequent active secretion [18], and is known to be induced in esophageal epithelial cells by weakly acidic bile salts [16]. In addition, the antioxidant properties of curcumin have been studied extensively in vitro and in vivo and may include direct superoxide scavenging and upregulation of scavenger enzymes [37]. Our findings agree with previous studies, demonstrating a higher level of oxidative stress in weakly acidic bile salt‐treated NE‐1 cells, which was ameliorated by curcumin pre‐treatment. Curcumin treatment has previously been investigated in the context of GORD, where 50 μM curcumin pre‐treatment reduced COX‐2 gene expression and NF‐κB phosphorylation in DCA‐treated esophageal epithelial cell lines, HET1A and OE33 [40, 41]. Intraduodenal administration of curcumin in a rat model of reflux oesophagitis has also shown significant improvement of histological signs of acute oesophagitis, such as neutrophil infiltration and interruption of the lamina propria [42]. Although Barrett's esophagus patients treated with oral curcumin did not significantly alter IL‐8 or I‐ΚB expression [41], increasing curcumin dosage, availability at the mucosal surface via topical application, and a sustained treatment remain to be evaluated [41]. Addition of 50 μM curcumin as a pre‐treatment in our study normalized HMGB1 release from NE‐1 cells to control levels [43] and was also associated with a decreased level of oxidative stress. Therefore, our study demonstrates that pre‐treatment of esophageal epithelial cells with curcumin can inhibit acidic bile salt–induced release of HMGB1, potentially through antioxidant activity. Further studies using human biopsies or in vivo perfusion of weakly acidic bile salt following topical treatment with curcumin or another antioxidant are required to assess the validity of this molecular pathway for therapeutic development, and the use of an immortalized cell line in a limitation of the present study. As bile salt‐induced oxidative stress is known to contribute to both inflammatory and metaplastic processes, targeting this pathway may have efficacy in both reflux oesophagitis and Barrett's esophagus.

Data presented here concurs with previously published studies describing the expression of TLR2 and TLR4 in healthy and GORD human esophagus [22, 44]. Similar to one previous study looking at both TLR2 and TLR4, we detected a greater percentage of epithelial cells positive for TLR4, compared to TLR2, across all patient groups [22]. However, while the previous study noted a higher level of TLR2 immunoreactivity in epithelial cells of patients with histological or macroscopic oesophagitis compared to healthy controls, but not TLR4, we found no difference in TLR2 but found TLR4 expression increased exclusively in ERD patients. One reason for this difference may be due to the patient stratification we used, where patients were categorized as FH, NERD, and ERD using clinical data points such as acid exposure time and presence of oesophagitis at the time of endoscopy. In contrast, the previously described study grouped patients based on biopsy histology only. Our use of clinical data to stratify patients into distinct groups reduces bias, as phenotypes are based on agreed‐upon clinical findings [27], as opposed to the level of histological inflammation present, which could be a confounding factor in the analysis. The impact of TLR2 and TLR4 activation by HMGB1 following a reflux event can be hypothesized. Activation of TLRs induces activation of NF‐κB‐mediated inflammatory pathways, resulting in the production of inflammatory mediators, which are known to be upregulated in GORD pathogenesis [45, 46]. Specific activation of TLR2 on esophageal cells derived from a murine Barrett's esophagus model induces release of CXCL‐1 and MIP‐2 [47]. Signaling through TLR4 induces cellular proliferation in HET1A cells via phosphorylation of NF‐κB [48], as well as inflammatory signaling in a Barrett's Esophagus cell line via COX‐2 [44]. Activation of these TLR pathways by HMGB1 in human esophageal mucosa could result in the release of inflammatory mediators, perpetuating acid‐induced inflammatory signaling. Release of such mediators could also activate deep and superficial CGRP+ sensory nerve fibers, resulting in heartburn sensation, as well as the lasting sensitization of these nerves.

We also observed a population of RAGE+CD45+ immune cells in the papillae of the esophageal mucosa, as well as infiltrating the epithelium. Although outside the scope of this study, future studies could utilize flow cytometry or other analytical methods to determine the exact subpopulations of leukocytes to which these cells belong. However, RAGE activation by HMGB1 or other ligands typically leads to downstream inflammatory pathways and polarization of leukocytes towards an inflammatory phenotype. For example, activation of RAGE on T cells promotes differentiation into Th1 and Th17 effector cells [49]. In dendritic cells, RAGE activation via HMGB1 induces production of cytokines including IL‐6 and IL‐8, as well as migration to lymph nodes [49]. Interestingly, CD1a+ dendritic cells are depleted in the esophageal mucosa of FH and ERD patients [28], which may be a result of such migration‐inducing mechanisms. We have also demonstrated the proximity of RAGE+ cells to CGRP+ nerve fibers in the esophageal mucosa. This could represent a mechanism by which sensing of noxious contents by epithelial cells, resulting in the release of molecules such as HMGB1, can indirectly activate or sensitize sensory nerve fibers through adjacent immune cells. Such interactions between immune cells and sensory neurons have been extensively described in skin [50]. Further, production of pro‐inflammatory cytokines in immune cells, including IL‐6, CXCL8, TNF‐α, and IL‐17, following RAGE activation may directly activate receptors on nearby sensory nerve fibers in the esophageal mucosa [49].

We have identified a novel molecular pathway associated with GORD pathogenesis, by demonstrating that HMGB1, released from esophageal epithelial cells in response to weakly acidic bile salts (at a pH relevant to patients on a PPI), has the potential to act on receptors localized within the esophageal epithelium, driving downstream activation of inflammatory signaling pathways within the mucosa. The cellular localisation of HMGB1 receptors, TLR2, TLR4, and RAGE, in the esophageal mucosa suggests a role in epithelial inflammation as well as indirect communication with CGRP+ sensory nerve fibers via closely apposed RAGE+ immune cells. These data suggest targeting antioxidant treatment strategies, topical or systemic, in GORD patients may alleviate symptoms by inhibiting HMBG1 release. Further studies are required to validate this specific pathway in GORD, for example investigating HMGB1 cellular localisation in vivo in esophageal biopsies following perfusion of acid and acidic bile salts, as well as investigating the therapeutic potential of topical anti‐oxidants, such as curcumin, in ameliorating downstream inflammation in response to weakly acidic bile salts in ex vivo esophageal biopsies.

## Author Contributions

T.L. conceived, designed, conducted experiments, and wrote the manuscript. P.W. contributed to experimental design and obtained funding. D.K. and D.B. contributed to experimental design, data curation, and edited the manuscript. M.P. conceived and designed experiments and edited the manuscript.

## Conflicts of Interest

The authors declare no conflicts of interest.

## Supporting information


Appendix S1.

## Data Availability

The sequencing data discussed in this publication are deposited in the NCBI's Gene Expression Omnibus and are accessible through the GEO Series accession number GSE226303.
