# Supplementary material for: A Role for High Mobility Group Box 1 (HMGB1) Release in the Pathogenesis of Gastroesophageal Reflux Disease
Source: Neurogastroenterol Motil. 2025 May 20;37(11):e70083. doi: 10.1111/nmo.70083 (PMC12534581; doi:10.1111/nmo.70083)
Supplement: Supplementary file 1 — Appendix S1. [file NMO-37-e70083-s001.docx]

**TITLE: A Role for High Mobility Group Box 1 (HMGB1) Release in the Pathogenesis of Gastro-oesophageal Reflux Disease**

**JOURNAL NAME:** Neurogastroenterology & Motility

**AUTHORS:** Mr Tom Leech^1^, Prof David Kelsell^2^, Dr Diana Blaydon^2^, Dr Philip Woodland^1^, Dr Madusha Peiris^1^

**AUTHOR AFFILIATIONS:**

1. Wingate Institute of Neurogastroenterology, Blizard Institute, The Faculty of Medicine and Dentistry, Queen Mary University of London, London, United Kingdom.
2. Centre for Cell Biology and Cutaneous Research, Blizard Institute, The Faculty of Medicine and Dentistry, Queen Mary University of London, London, United Kingdom.

**CORRESPONDING AUTHOR:** Dr Madusha Peiris, Wingate Institute of Neurogastroenterology, 26 Ashfield Street, London, E1 2AJ. m.peiris@qmul.ac.uk. +44 207 882 2634.


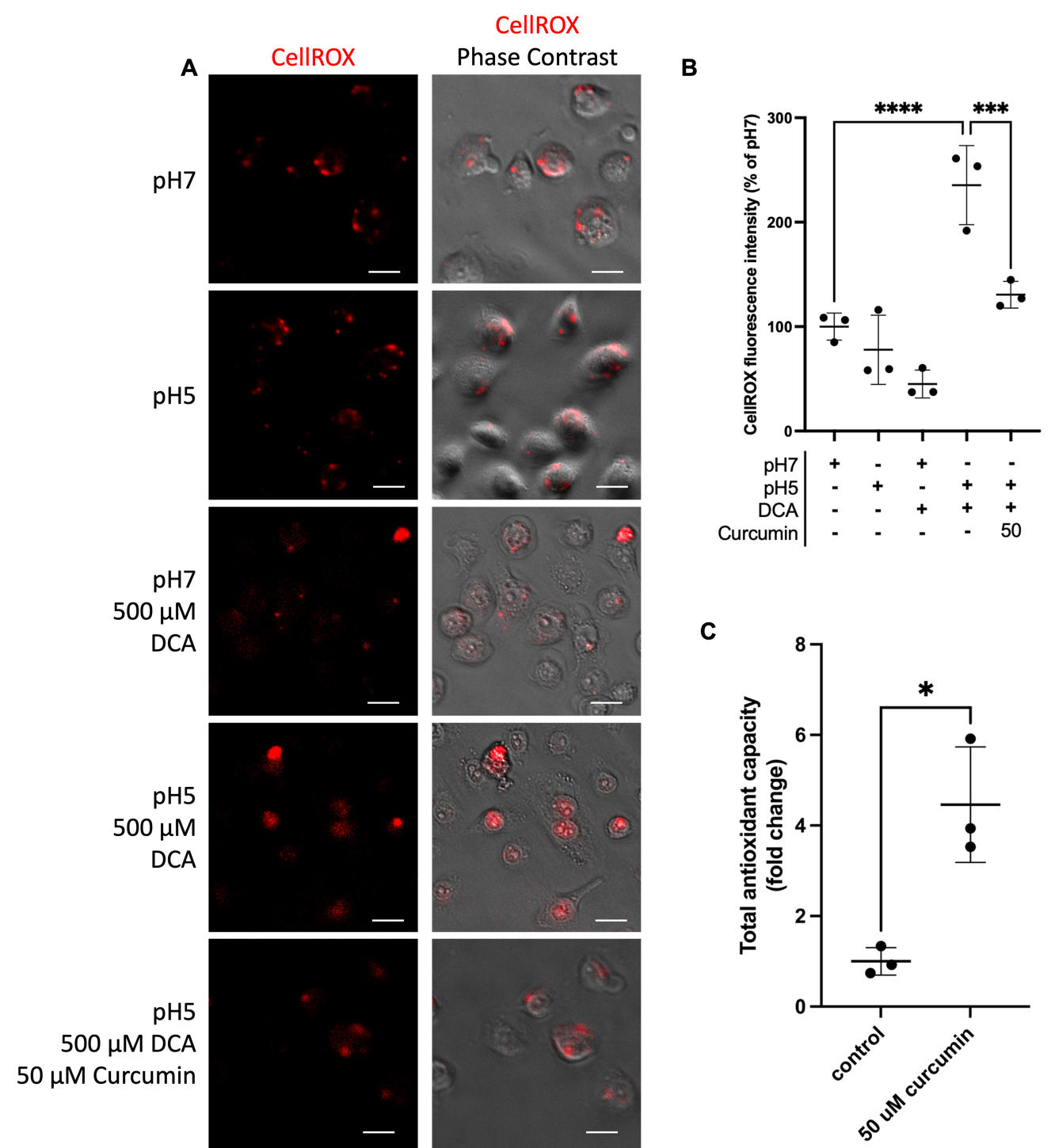


**Supplementary figure 1.** (**A**) Representative images showing CellROX™️ fluorescence in NE-1 cells following a 10 minute incubation with a combination of weak acid (pH5) and 500 µM Deoxycholic acid (DCA). Curcumin pre-treated cells were incubated for 2 hours prior to acidic bile salt incubation. (**B**) Fluorescence intensity of CellROX™️ staining in NE-1 cells. Presented as mean ± SD. ∗∗∗*P* < .001, ∗∗∗∗*P* < .0001. n = 3. (**C**) Total antioxidant capacity of NE-1 cells after 2-hour incubation with normal media or media containing 50 μM curcumin (p = 0.0103; n=3). Presented as mean ± SD fold-change compared to control cells.


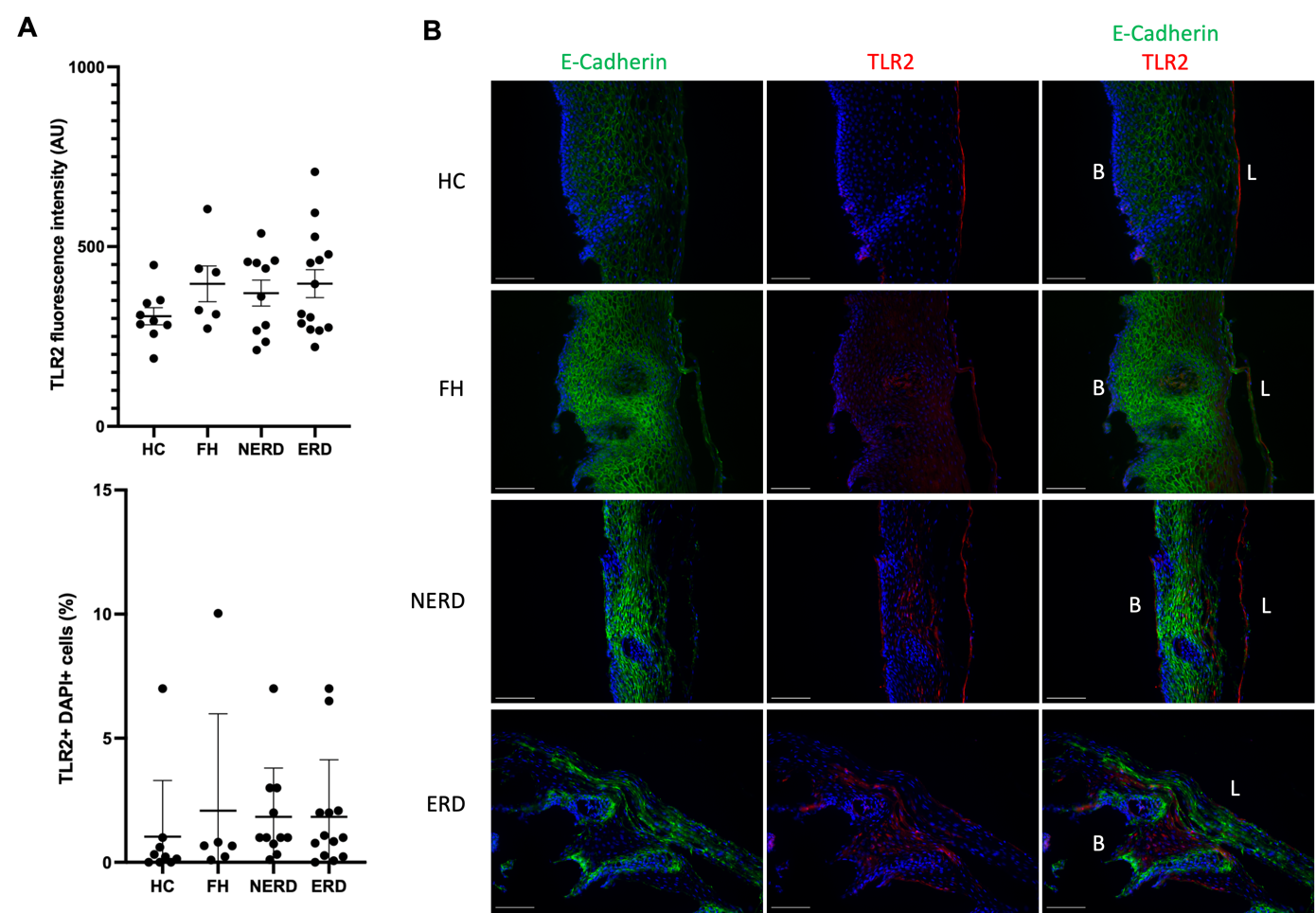


**Supplementary figure 2**. Quantification of TLR2 in the oesophagus of healthy controls and GORD patients. (A) Mean fluorescence intensity of TLR2 signal in the oesophageal epithelium and % of DAPI+ cells which were TLR2+. (B) Representative images of TLR2 in the oesophageal mucosa. Mean ± SD. L = luminal side of epithelium. B = basal side of the epithelium. Images were captured with a Leica DM5000B Epi-Fluorescence Microscope and a DFC350 FX camera using MetaMorph Software. Quantification of protein expression or staining was performed using ImageJ (National Institutes of Health).

| **Functional Heartburn** | | | | |
| --- | --- | --- | --- | --- |
| **Gene Name** | **Mean Expression (TPM)** | **log2FoldChange** | **P value** | **Adjusted P value** |
| RNASET2 | 22.3 | 1.96 | 0.000177 | 0.0277 |
| **Non-erosive Reflux Disease** | | | | |
| **Gene Name** | **Mean Expression (TPM)** | **log2FoldChange** | **P value** | **Adjusted P value** |
| SELENOP | 84.4 | 1.96 | 0.00000000468 | 0.00000364 |
| TNFSF10 | 94.2 | 1.73 | 0.0000000275 | 0.0000107 |
| UQCRB | 222 | 1.46 | 0.00000366 | 0.00057 |
| PPIA | 2160 | 1.35 | 0.00000786 | 0.00102 |
| ATP5PF | 180 | 1.46 | 0.0000495 | 0.00372 |
| NDUFA1 | 94.1 | 1.53 | 0.0000573 | 0.00372 |
| NDUFA4 | 57.9 | 1.59 | 0.0000537 | 0.00372 |
| ATP5MK | 631 | 1.39 | 0.000113 | 0.00584 |
| IDH1 | 79.2 | 1.46 | 0.000109 | 0.00584 |
| CPT1B | 11.9 | 2.13 | 0.000152 | 0.00588 |
| NDUFAB1 | 62.8 | 1.42 | 0.000151 | 0.00588 |
| NPM1 | 628 | 1.38 | 0.000166 | 0.00588 |
| PSMA6 | 136 | 1.46 | 0.000135 | 0.00588 |
| RPS27A | 1050 | 1.35 | 0.000139 | 0.00588 |
| UQCRQ | 226 | 1.39 | 0.000163 | 0.00588 |
| AKR1C3 | 30.3 | 2.10 | 0.000662 | 0.0191 |
| GSTA4 | 64.4 | 1.51 | 0.000881 | 0.0221 |
| OMA1 | 14.5 | 1.70 | 0.00114 | 0.0247 |
| HMGB1 | 323 | 1.40 | 0.00126 | 0.0261 |
| HSP90AA1 | 283 | 1.43 | 0.00127 | 0.0261 |
| ARL6IP5 | 44.9 | 1.35 | 0.00137 | 0.0266 |
| TIMP1 | 13.9 | 2.16 | 0.00136 | 0.0266 |
| PPIG | 47.6 | 1.57 | 0.00148 | 0.0281 |
| CD47 | 86.3 | 1.41 | 0.00234 | 0.038 |
| MT-CO3 | 8070 | 1.31 | 0.00263 | 0.041 |
| GLO1 | 51.7 | 1.33 | 0.00276 | 0.0422 |
| **Erosive Reflux Disease** | | | | |
| **Gene Name** | **Mean Expression (TPM)** | **log2FoldChange** | **P value** | **Adjusted P value** |
| NPM1 | 642 | 1.44 | 0.000000317 | 0.000122 |
| PRNP | 28.3 | 2.04 | 0.00000367 | 0.000369 |
| TIMP1 | 21.8 | 3.79 | 0.00000382 | 0.000369 |
| DDIT4 | 72.8 | 1.93 | 0.000012 | 0.000924 |
| HMGB1 | 315 | 1.35 | 0.0000159 | 0.000977 |
| PPIA | 2180 | 1.38 | 0.0000154 | 0.000977 |
| HSP90AA1 | 293 | 1.52 | 0.0000353 | 0.00184 |
| PCNA | 62.4 | 1.43 | 0.0000803 | 0.00295 |
| SELENOP | 71.3 | 1.52 | 0.0000993 | 0.00295 |
| YBX1 | 350 | 1.37 | 0.0000981 | 0.00295 |
| ATP5PF | 176 | 1.41 | 0.000116 | 0.00299 |
| GJA1 | 68.5 | 1.68 | 0.000115 | 0.00299 |
| SLPI | 1810 | 2.14 | 0.00011 | 0.00299 |
| CAV1 | 17.4 | 2.32 | 0.00029 | 0.00559 |
| SCD | 80.6 | 1.96 | 0.000339 | 0.00639 |
| FUS | 127 | 1.39 | 0.000395 | 0.00725 |
| PPIG | 48.3 | 1.60 | 0.000471 | 0.00826 |
| TPM1 | 12.8 | 2.02 | 0.000576 | 0.00907 |
| CASP1 | 50.3 | 1.66 | 0.000648 | 0.01 |
| AKR1B1 | 14 | 2.23 | 0.000713 | 0.0107 |
| HSPA8 | 613 | 1.36 | 0.000807 | 0.0115 |
| ALDH7A1 | 57.7 | 1.34 | 0.00103 | 0.0139 |
| PARK7 | 123 | 1.43 | 0.00102 | 0.0139 |
| KRT5 | 3970 | 1.43 | 0.00121 | 0.0151 |
| NDUFAB1 | 63.1 | 1.44 | 0.00124 | 0.0152 |
| GSTA4 | 65.7 | 1.57 | 0.00126 | 0.0152 |
| NME1 | 81.3 | 1.49 | 0.00131 | 0.0156 |
| PSMA6 | 136 | 1.46 | 0.00137 | 0.0157 |
| SLC3A2 | 72 | 1.48 | 0.00162 | 0.0178 |
| BAK1 | 16.7 | 1.77 | 0.00182 | 0.0196 |
| NDUFAF2 | 37.3 | 1.45 | 0.00189 | 0.02 |
| VIM | 42 | 1.72 | 0.00231 | 0.0231 |
| NDUFA4 | 54.1 | 1.43 | 0.00234 | 0.0231 |
| APP | 55.3 | 1.63 | 0.00239 | 0.0234 |
| BAX | 44.5 | 1.44 | 0.00262 | 0.0246 |
| RPS27A | 1030 | 1.31 | 0.00352 | 0.0316 |
| C1QBP | 115 | 1.29 | 0.00384 | 0.0333 |
| FOS | 12.2 | 3.72 | 0.00418 | 0.0359 |
| CYCS | 138 | 1.35 | 0.00462 | 0.0383 |
| TOP2A | 21.8 | 1.64 | 0.0047 | 0.0386 |
| MRPS16 | 39 | 1.39 | 0.00557 | 0.0448 |
| YWHAQ | 226 | 1.37 | 0.00595 | 0.0473 |
| CCNB1 | 40.1 | 1.56 | 0.00607 | 0.0478 |

**Supplementary table 1**. Oxidative stress –related genes which are upregulated in FH, NERD, or ERD compared to healthy controls in bulk RNA sequencing. 1399 oxidative stress protein domains were extracted from GeneCards (https://www.genecards.org) with a relevance score ≥ 7. Differential expression analysis (DEseq2) was performed between healthy controls and functional heartburn (FH) patients, healthy controls and non-erosive reflux disease (NERD) patients, as well as healthy controls and erosive reflux disease (ERD) patients, with an false discovery rate (FDR) adjusted P value filter of <0.05. Genes with a log2 fold change (FC) < 1 or an average transcripts per million (TPM) count of < 10 were removed. A total of 1 oxidative stress-associated gene was upregulated in FH, 26 in ERD, and 43 in ERD.
